# Multidrug resistance protein 1 reduces the aggregation of mutant huntingtin in neuronal cells derived from the Huntington’s disease R6/2 model

**DOI:** 10.1038/srep16887

**Published:** 2015-11-20

**Authors:** Wooseok Im, Jae-Jun Ban, Jin-Young Chung, Soon-Tae Lee, Kon Chu, Manho Kim

**Affiliations:** 1 Department of Neurology, Seoul National University Hospital, Seoul, South Korea; 2 Department of Veterinary Internal Medicine and Geriatrics, College of Veterinary Medicine, Kangwon National University, Chuncheon, South Korea; 3 Protein Metabolism Medical Research Center, College of Medicine, Seoul National University, Seoul, South Korea

## Abstract

Mutant huntingtin (mHtt) aggregation in the nucleus is the most readily apparent phenotype and cause of neuronal death in Huntington’s disease (HD). Inhibiting mHtt aggregation reduces cell death in the brain and is thus a promising therapeutic approach. The results of the present study demonstrated that mHtt aggregation in the nucleus was altered by the activity of multidrug resistance protein 1 (MDR1), which was experimentally modulated by verapamil, siRNA and an expression vector. MDR1 detoxifies drugs and metabolites through its excretory functions in the membrane compartment, thereby protecting cells against death or senescence. When they were treated with verapamil, R6/2 mice showed a progressive decline in rotarod performance and increased mHtt aggregation in the brain. Using neuronal stem cells from R6/2 mice, we developed an *in vitro* HD model to test mHtt accumulation in the nuclei of neurons. When MDR1 activity in cells was decreased by verapamil or siRNA, mHtt aggregation in the nuclei increased, whereas the induction of MDR1 resulted in a decrease in mHtt aggregation. Thus, our data provide evidence that MDR1 plays an important role in the clearance of mHtt aggregation and may thus be a potential target for improving the survival of neurons in Huntington’s disease.

Huntington’s disease is a progressive, neurodegenerative disorder that is caused by the expansion of a CAG repeat in the *huntingtin* gene and results in the production of the mutant polyglutamine (PolyQ)-expanded huntingtin protein (mHtt)^1^. mHtt accumulates and forms intracellular aggregates in the nuclei of cells as the disease progresses. mHtt has been implicated in several processes that can cause cell death, such as mitochondrial dysfunction, transcriptional dysregulation, altered protein-protein interactions, abnormal protein aggregations, and excitotoxicity^2^,^3^,^4^.

Multidrug resistance-1 (MDR1) is a P-glycoprotein (Pgp) and an ATP-binding cassette sub-family B member 1 (ABCB1). Murine MDR1 is encoded by the mdr1a and mdr1b genes. These genes have 90% sequence homology to one another and have 80% homology to the human gene^5^. MDR1 is important for excretory functions and is widely expressed in normal tissues, such as the liver, kidney, intestine, and the blood-brain barrier^6^,^7^,^8^,^9^. The primary cellular function of MDR1 is detoxification by transportation^10^. MDR1 has been studied intensively in various cancers because cancer cells highly express this protein. Moreover, it has been reported that beta-amyloid proteins are transported from the brain into the blood through MDR1^11^,^12^. MDR1 is also expressed in the cells of the nervous system, including neurons, astrocytes, microglia, and oligodendrocytes. MDR1 resides in the plasma membrane and the membranes of intracellular compartments, including the Golgi, endosomes, and the mitochondria^6^,^8^,^13^. A recent study has demonstrated that MDR1 interacts with PolyQ and can directly affect PolyQ inclusion in *Drosophila*^14^. Yet, whether MDR1 affects mHtt aggregation in HD is unknown.

In the present study, we investigated whether MDR1 inhibits the aggregation of mHtt using R6/2 transgenic HD mice (R6/2) that express exon 1 of the human huntingtin gene with >110 CAG repeats and display mHtt aggregation in the cells of the brain^15^. R6/2 mice were treated with verapamil, which is an inhibitor of MDR1, and were then tested for rotarod performance to determine whether lowering the activity of MDR1 affects the progression of HD. Neuronal stem cells from R6/2 mice were used to study the effects of MDR1 on mHtt aggregation at the cellular level because MDR1 is strongly expressed in neural stem cells^16^,^17^,^18^ and because its expression is downregulated when these cells are differentiated^19^,^20^,^21^. In addition, the accumulation of mHtt was analysed in the nucleus after inducing the expression of MDR1 in cells.

## Results

### Verapamil accelerated a decline in the rotarod performance of R6/2 mice

**Figure 1:**
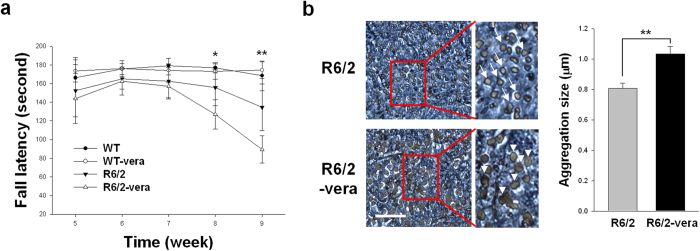
Rotarod performance and immunohistochemistry of mHtt aggregation in R6/2 mice that were treated with verapamil. (**a**) Rotarod performance of the R6/2-vera group was lower at weeks eight and nine when compared to those of the controls (R6/2). R6/2 vs R6/2-vera: *F*_*3,16*_ = 1.65, *P* = 0.22 at five weeks; *F*_*3,16*_ = 2.10, *P* = 0.14 at six weeks; *F*_*3,16*_ = 3.05, *P* = 0.06 at 7 weeks; *F*_*3,16*_ = 14.53, *P* = 0.032 at 8 weeks; *F*_*3,16*_ = 25.87, *P* = 0.0082 at 9 weeks. (**b**) The size of the Em48-stained population (arrows and arrowheads) was much larger in R6/2-vera mice than in R6/2-control (R6/2) mice at week 9. *P < 0.05, **P < 0.01, scale bar = 10 μm.

Verapamil, which is used for its MDR-reversal effect in mice^22^, was orally administered every day from three to nine weeks. Rotarod tests were performed from the fourth to the ninth week (n = 4 for wild type, WT; n = 4 for wild type treated with verapamil, WT-vera; n = 5 for R6/2 control, R6/2; and n = 5 for R6/2 mice treated with verapamil, R6/2-vera). R6/2 rotarod performance was impaired compared to the wild type mice (*F*_*1,48*_ = 13.93*; P* < 0.001), and treatment with verapamil did not affect the performance of the wild type mice (Fig. 1a). Conversely, verapamil decreased the rotarod performance of R6/2 mice at weeks eight (*F*_3,16_ = 14.53, *P* = 0.032) and nine (*F*_3,16_ = 25.87, *P* = 0.0082; Fig. 1a). Following the rotarod test at week nine, the brains were isolated and assessed for the accumulation of mHtt aggregation. Verapamil treatment augmented the accumulation of mHtt aggregation (arrows and arrowheads) in the striata of treated mice compared with those of the control mice (Fig. 1b and Fig. S1).

### Differentiated neural stem cells from R6/2 transgenic mice (R6/2-NSC) displayed an accumulation of mHtt aggregates, and MDR1 was downregulated following R6/2-NSC differentiation (dif-R6/2-NSC)

**Figure 2:**
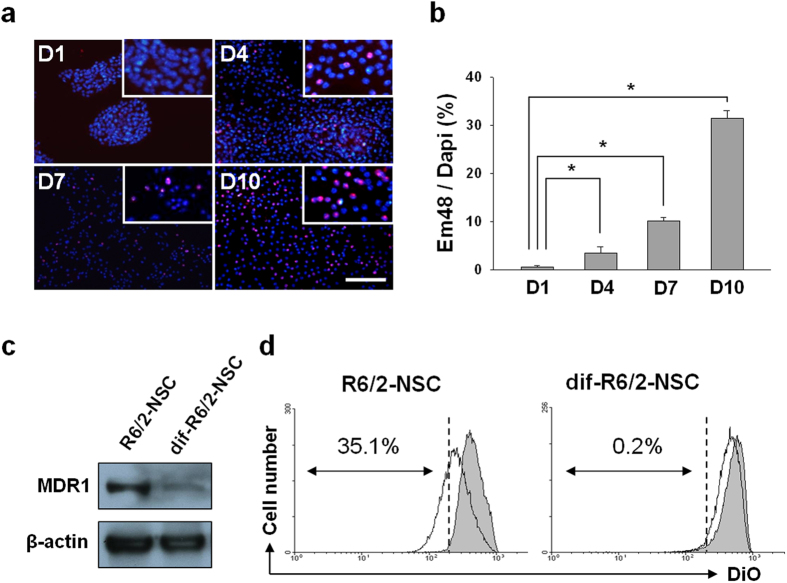
Investigation of mHtt aggregations and MDR1 activity in R6/2-NSC after differentiation. (**a**) R6/2-NSC were stained with Em48 (mHtt, red) and DAPI (nucleus, blue) at days one, four, seven, and. ten after differentiation. (**b**) Ratios of the Em48 (+) to DAPI (+) cells increased with time. (**c**) Western.blot results confirmed that the expression of MDR1 decreased in dif-R6/2-NSC compared to R6/2-NSC. Full length blots are shown in the supplementary data (Fig. S7a). (**d**) R6/2-NSC or dif-R6/2-NSC were. incubated with DiOC2(3) and treated with vinblastine (grey) or DMSO (blank). Cells incubated at 37 ºC. with DMSO (diluents control) have low fluorescence, indicating that MDR1 effluxes the dye, whereas. cells show high fluorescence in the presence of vinblastine, a competitive MDR1 inhibitor. The. fluorescence of the remaining DiOC2(3) (DiO) was measured by flow cytometry. R6/2-NSC showed a. reduction of 35.1%, whereas dif-R6/2-NS show only a 0.2% reduction. *P < 0.001, scale bar = 50 μm.

R6/2-NSC displayed mHtt aggregation following the induction of differentiation. The aggregates were detected using the Em48 antibody at one, four, seven, and ten days following differentiation (Fig. 2a). The number of cells that were positive for staining with the Em48 antibody increased in a time-dependent manner, with the highest level of staining occurring at day 10 (Fig. 2b). The expression and activity of MDR1 were investigated in R6/2-NSC and dif-R6/2-NSC. Western blotting showed that MDR1 was expressed in R6/2-NSC but was downregulated in dif-R6/2-NSC (Fig. 2c). It has been reported that MDR1 is downregulated when neural stem cells differentiate^19^,^20^,^21^. To confirm the decrease of activity of MDR1 in dif-R6/2-NSC, we measured the intracellular accumulation of DiOC_2_(3), a fluorescent MDR1 substrate. The results showed that the intracellular accumulation of DiOC_2_(3) decreased inside of R6/2-NSC compared with the control (Fig. 2d left); however, no difference was observed between the dif-R6/2-NSC and control cells (Fig. 2d right), which indicates that MDR1 activity decreased in dif-R6/2-NSC. These results suggest that the accumulation of mHtt aggregation can be correlated with the activity of MDR1.

### Lowering the activity of MDR1 increased the accumulation of mHtt aggregation

**Figure 3:**
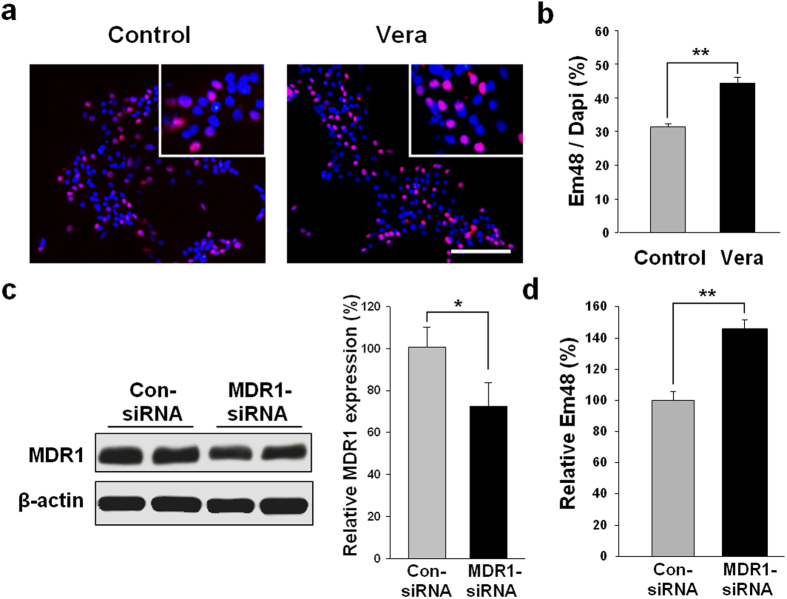
Investigation of mHtt aggregation in R6/2-NSC in which the activity or expression of MDR1 was decreased by verapamil and MDR1 siRNA. (**a**) R6/2-NSC were treated with verapamil (20 μm) after day two of differentiation and stained with the Em48 antibody at day ten of differentiation. (**b**) The ratios of Em48 (+) to DAPI (+) cells were calculated. The verapamil-treated group (44.4 ± 3.2%) showed an increase in mHtt aggregation compared to the control group (31.3 ± 1.9%). Dif-R6/2-NSC were transfected with MDR1 siRNA or control siRNA as a control, and the levels of MDR1 were measured by western blot (**c**). Full length blots are shown in the supplementary data (Fig. S7b). The numbers of mHtt aggregations were also investigated at day seven by immunocytochemistry (**d**). Transfection of MDR1 siRNA increased the accumulation of mHtt aggregation. (*P < 0.05, **P < 0.01), Scale bar = 100 μm

To determine whether MDR1 was related to mHtt accumulation, we inhibited the activities of MDR1 with verapamil. Verapamil (20 μM) was administered from days two to ten following the differentiation of R6/2-NSC. At day 10, the control and verapamil groups (Vera) were fixed with 4% paraformaldehyde. The cells were stained with the Em48 antibody and DAPI and visualised using immunofluorescence (Fig. 3a). We counted DAPI (+) cells and compared this count to that of the dual DAPI (+) and Em48 (+) cells for each group. The ratios of the double-positive cells to DAPI (+) cells were 31.3 ± 1.9% for the control groups and 44.4 ± 3.2% for the verapamil groups (Fig. 3b).

We next investigated mHtt aggregation following the siRNA-mediated reduction of *Mdr1* mRNA in dif-R6/2-NSC. MDR1 or control siRNA was transfected into dif-R6/2-NSC, and western blots were used to confirm the decreased expression of MDR1 in cells (Fig. 3c). Five days following transfection, mHtt was also evaluated by immunocytochemistry with the Em48 antibody and DAPI. The ratio of double-positive to DAPI (+) cells was 18.1 ± 0.8% for cells transfected with MDR1 siRNA and 12.4 ± 1.0% for cells transfected with control siRNA. These results demonstrated a 46.0% increase in the level of mHtt aggregation (+) cells after MDR1 knockdown using specific siRNA (Fig. 3d).

### Overexpression of MDR1 by the pCMV6-MDR1-GFP plasmid prevented mHtt accumulation

**Figure 4:**
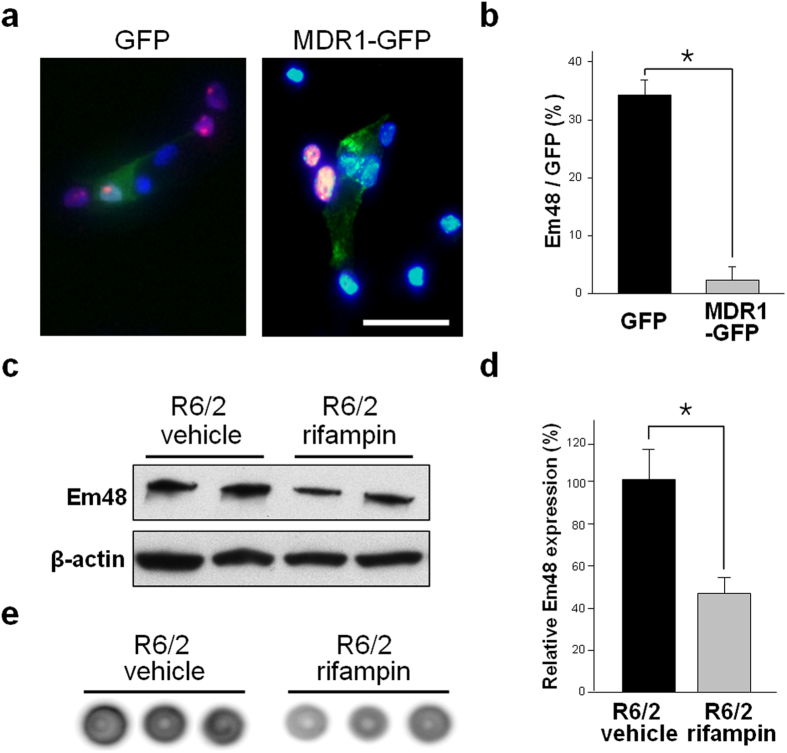
Activation of MDR1 in *in vitro* or *vivo* HD models. (**a**) MDR1-GFP or GFP plasmids (green) were transfected into dif-R6/2-NSC at day two, and the cells were stained with Em48 (red) and DAPI (blue) at day eight. (**b**) Cells containing the MDR1-GFP plasmid (black box) showed 2.5 ± 2.5% Em48 and GFP (+) cells, whereas the control GFP groups (grey box) had 34.3 ± 2.5% Em48 (+) cells. (**c**) Western blot analysis showed that rifampin decreased mHtt accumulation in R6/2 mice. Full length blots are shown in the supplementary data (Fig. S7c). (**d**) Bar graphs show the relative levels of protein expression, normalised to the control (R6/2 vehicle). (**e**) A dot blot assay confirmed that rifampin decreased mHtt accumulation in R6/2 mice. All dot blots are shown in the supplementary data (Fig. S7e-g) *P < 0.01, scale bar = 5 μm.

We investigated the accumulation of mHtt aggregations following the overexpression of the MDR1 protein using the pCMV6-MDR1-GFP plasmid (MDR1 plasmid) or pCMV6-GFP plasmid (GFP plasmid). Dif-R6/2-NSC were transfected with the MDR1 or GFP plasmid, and mHtt aggregation was evaluated using immunocytochemistry with Em48 antibody and DAPI. Fluorescence-positive cells, such as MDR1-GFP and GFP only (green), Em48 (red) and DAPI (blue), were observed with a fluorescence microscope (Fig. 4a). Dif-R6/2-NSC transfected with the MDR1 plasmid expressed 2.5 ± 2.5% Em48 (+) cells, while the control GFP groups expressed 34.3 ± 2.5% Em48 (+) cells of total GFP positive cells (Fig. 4b and Fig. S2).

### Rifampin treatment reduced mHtt accumulation in R6/2 mice

Rifampin, which is a potential inducer of MDR1^12^,^23^, was intraperitoneally administered to R6/2 mice daily from five to twelve weeks. To investigate mHtt accumulation, we extracted proteins from whole brains of R6/2 mice that were administered vehicle (DMSO) or rifampin and then measured protein levels using western blots and dot blot assays (Fig. 4c,d,e and S7). Rifampin decreased mHtt accumulation in the brain of R6/2 when compared with controls.

## Discussion

The purpose of the present study was to determine whether MDR1 could affect the accumulation of mHtt aggregates in experimental models of Huntington’s disease. Verapamil and MDR1 siRNA increased mHtt accumulation in an *in vitro* HD model, and the former decreased the performance of R6/2 transgenic mice in the rotarod test. The overexpression of MDR1 using an MDR1 plasmid decreased the accumulation of mHtt aggregation in the *in vitro* HD model. Taken together, these results indicate that MDR1 has an important role in modulating the accumulation of mHtt aggregation in cells.

R6/2 transgenic mice are widely used for investigating HD and its pathological phenotypes, such as body weight decrease and motor impairment, which are correlated with the CAG repeat length^24^. We used F1 pups of R6/2 mice for *in vivo* tests and assessed the number of CAG repeats in each group (Fig. S6). HD BAC transgenic mice display a body weight increase that influences their rotarod test results^25^. Additionally, the decrease in the body weight of R6/2 mice is accompanied by motor impairment^15^. The verapamil treatment group demonstrated early motor impairment, but body weight was unaffected (Fig. S3a) in R6/2 mice. Although treatment with rifampin, which induces the activation of MDR1^23^, quantitatively reduced the nuclear accumulation of mHtt aggregates, these results were observed in the brain tissue via immunofluorescence (Fig. S4) and did not correlate with an improvement in the behavioural functions of the R6/2 mice (Fig. S3b). We speculated that when MDR1, a transporter protein of the cell membrane, is activated by rifampin, it releases polyQ fragments out of the cytosol and results in decreased mHtt aggregations in cells; however, the nuclear aggregates were not completely eliminated. Therefore, rifampin did not improve the disease progression in R/2 mice. However, the MDR1 inhibitor verapamil increased the accumulation of mHtt aggregates in cells, thereby resulting in the rapid behavioural decline of R6/2 mice. We suggest that further investigations using other HD mouse models that display long-term phenotypes, such as the BAC and zQ715 mice^26^,^27^, are needed because the effects of rifampin might be more notable in these models. In addition, the exact mechanism by which polyQ fragments are cleared by MDR1 is presently unknown.

We observed that the effect of verapamil on mHtt aggregation is time dependent (Fig. S5). The results demonstrated that treatment with verapamil at an early time point influenced the aggregation of mHtt. However, the aggregation status was unchanged when treated at a later time point. This result implies that MDR1 affects mHtt aggregation at an early stage. Thus, the initial aggregation of the cytosolic CAG fragment, which is a component of mHtt aggregates, can be modulated by MDR1, thereby promoting cell survival by decreasing toxic aggregates. Furthermore, most cells have nuclear aggregates in *in vitro* and *in vivo* HD models, and very few cells demonstrated perinuclear aggregates in culture. Rifampin-treated groups displayed condensed spots of Em48 staining inside the nucleus, while the control group had smear staining that surrounded the condensed spot in the nucleus (Fig. S4). We speculate that MDR1 activation by rifampin might be related to the smear of Em48 staining around the nucleus.

Aggregation of mHtt has been proposed to account for the pathogenesis of Huntington’s disease by inducing cytotoxicity and neuronal death^3^,^28^,^29^,^30^,^31^. The aggregation of mHtt or of its metabolites increases in a polyQ-dependent manner and is associated with cell senescence^32^. The N-terminal mHtt fragments are toxic and are more likely to aggregate than the intact protein. Therefore the inhibition of proteolytic enzymes, such as caspases^33^,^34^,^35^ or calpain^36^,^37^, has been proposed as a mechanism by which aggregation can be reduced. Compounds^38^,^39^,^40^,^41^ such as Congo red^38^ and trehalose^39^ that modulate chaperones^40^ or small heat-shock proteins^41^ have been reported to inhibit toxic aggregation. Because enhancing transport may reduce accumulation, MDR1 is a novel candidate for facilitating the clearance of mHtt^42^,^43^. It is known that mHtt also represses the transcription of MDR1^44^. Therefore, we posited that MDR1 dysfunction is related to the progressive accumulation of mHtt aggregates.

Probenecid, a non-selective inhibitor of MDRs, improved the survival of N171-82Q transgenic mice^45^. Behavioural open field tests and histology demonstrated that probenecid administration reduced the loss of neurons in the striatum, thereby resulting in improved functional and survival outcomes. This indicates that the increased transport of toxic metabolites has beneficial effects^45^. Contrary to this previous finding, the inhibition of MDRs by verapamil worsened the behavioural and pathological outcomes. Therefore, both the inhibition and activation of MDR1 confirmed that MDR1 is associated with the intracellular accumulation of mHtt aggregates.

In this study, we utilised the neural stem cells of R6/2 transgenic mice to test MDR1 and mHtt accumulation^46^. MDR1 is highly enriched in neuronal stem cells but is known to be downregulated when mHtt aggregation increases in cells following differentiation from R6/2-NSC^20^,^21^,^47^. Therefore, differentiated R6/2-NSC might be an effective model in which to investigate the relationship between mHtt aggregation and MDR1 activity. The worsening behaviour and histology of R6/2 transgenic mice following verapamil treatment further supports the functional significance of MDR1 *in vivo*. However, the observation was limited up to the ninth week of experiments because mHtt accumulation could not be compared due to saturation effects. In more advanced stages beyond this point, however, an MDR1 assay conducted at week 12 demonstrated lower MDR1 activity than that observed at week eight (data not shown).

MDR1-specific inhibition by siRNA also replicated the alteration of mHtt in this study. Further studies, such as targeting genes or a drug delivery system to activate MDR1 *in vivo*, are warranted. Most neurodegenerative disorders, such as Alzheimer’s disease (AD), Parkinson’s disease (PD), Huntington’s disease (HD), amyotrophic lateral sclerosis (ALS), and prion diseases, share a common pathology of protein aggregation and inclusion body formation in the central nervous system at sites of neuronal degeneration^29^,^48^. The increase in aggregation or inclusion body formation might be caused by an increasing protein concentration, while the benign stage prior to visible protein aggregation might be related to the pathogenesis of the condition^48^. Releasing toxic proteins from cells can serve to protect cells against death by reducing the aggregation of abnormal proteins.

In conclusion, our results demonstrate that mHtt aggregation might be regulated by MDR1, which suggests that MDR1 might be a potential therapeutic target for Huntington’s disease.

## Methods

### Ethics statement

All animal experiments were conducted in accordance with the National Institutes of Health guide for the care and use of laboratory animals (NIH Publications No. 8023, revised 1978). Animals were kept under a 12 h light-dark cycle with food and drinking water. All animal studies were approved by the Institutional Animal Care and Use Committee (IACUC) at Seoul National University Hospital.

### Behavioural tests in R6/2 transgenic Huntington’s disease mice

Huntington’s disease transgenic mice of the R6/2 line (B6CBA-Tg(HDexon1)62Gpb/3J) were purchased from The Jackson Laboratory (Fig.S6). R6/2 mice received verapamil orally (mean dose of 3.5 mg/mouse per day) in their drinking water from the fourth to ninth weeks of age^49^. The groups were divided into verapamil-treated R6/2 mice (n = 5; 3 males and 2 females), vehicle-treated R6/2 mice (n = 5; 3 males and 2 females), control wild type mice (n = 4; 2 males and 2 females males), and verapamil-treated wild type mice (n = 4; 2 males and 2 females). For the induction of MDR1, R6/2 mice were injected with rifampin (n = 4 males, 100 mg/kg i.p.) or DMSO (n = 4 males, 4 μl/g i.p.) as a vehicle every day from weeks five to twelve^23^, and wild type mice (n = 4 males) served as the control group. Rotarod performance was assessed as previously described^50^ but with a modification to the accelerating rotarod (Jungdo Instruments, Seoul, Korea), which was set to linearly increase the speed from 4 to 40 rpm over three min. At four weeks of age, mice were trained on three consecutive days for three trials per day with a rest period of approximately 30 min between trials. Mice were tested for three trials once a week, and the mean latencies to fall were used for statistical analyses.

### Immunohistochemistry

Mice were anesthetised using 1.6 μl/g Zoletil (Verbac Laboratories, Carros, France) and 0.05 μl/g Rompun (Bayer) and perfused with 10 mL of cold saline and 10 mL of 4% paraformaldehyde in 0.1 M PBS at nine weeks of age. Brains were removed, cryoprotected in 30% sucrose at 4 °C, and sectioned into 20-μm slices^51^. Sections were stained with mouse anti-Em48, rinsed, and incubated in avidin–biotin complex (Vector Laboratories, Burlingame, CA, USA) for 2 h at 4 °C. Then, sections were rinsed and incubated in 3,3′-diaminobenzidine (DAB) and hydrogen peroxide solutions (Vector Laboratories, Burlingame, CA, USA). They were rinsed and mounted on glass slides, and coverslips were placed on the slides. The size of each aggregation stained with DAB was measured and quantified using Leica Application Suite (Leica Company, Switzerland). Sites (n = 4, each) were randomly selected in the defined boundary of the striatum.

### Primary culture of neural stem cells from R6/2 mice (R6/2-NSC)

To isolate neural stem cells from a striatum of the neonatal brain, we used a modified protocol by Reynolds and Weiss^16^. Briefly, brain tissues of mice at nine weeks of age were dissected in 35-mm petri dishes containing HBSS and were then mechanically minced in trypsin (Sigma, St. Louis, MO, USA). After 15 min of incubation at room temperature, tissues were triturated in Dulbecco’s modified Eagle’s medium (DMEM)/F12 (Gibco BRL). Cells were centrifuged at 200 × *g* for 15 min and seeded into 6-well plates in a culture medium consisting of DMEM/F12, 1% PSA (penicillin-streptomycin-amphotericin; Invitrogen, Carlsbad, CA, USA), 2% B27 Supplement (Gibco BRL), 10 ng/mL epidermal growth factor (EGF; Invitrogen) and 10 ng/mL basic fibroblast growth factor (bFGF; Invitrogen). Cells were incubated at 37 °C in a 95% O_2_ and 5% CO_2_ humidified atmosphere. Fresh culture medium was added every four to five days.

**Differentiation of R6/2-NSC.** R6/2-NSC were differentiated in differentiation culture media composed of DMEM/F12, 1% PSA, 2% B27, and 4% FBS^18^,^52^,^53^. Cells were cultured for 4–5 days in the culture medium, after which the old culture medium was removed and cells were cultured in differentiation culture medium.

**Quantification of mHtt accumulation.** To quantify the degree of mHtt aggregation, cells were assessed by fluorescent immunocytochemistry. Cells were incubated on poly-L-lysine–coated coverslips. Cells were fixed with 4% paraformaldehyde in 0.1 M PBS for 15 min and blocked in 4% normal goat serum and 0.2% Triton X-100 in PBS for 1 h. Cells were then incubated overnight with Em48 antibody (Millipore, 1:400), which detects the polyQ domain of mutant huntingtin protein, followed by staining with Cy5-conjugated secondary antibody for 2 h. The cells were additionally counterstained with DAPI to label cell nuclei. The number of Em48 (red)- or DAPI (blue)-stained cells was counted and averaged from five randomly selected microscopic fields at 100× magnification viewed with an inverted microscope (BX61, Olympus Corporation, Tokyo, Japan).

### Analysis of MDR1

To investigate the expression and function of MDR1, we performed western blot analysis and an MDR efflux assay. To directly measure the functional activity of MDR1, the ability of the cell to extrude fluorescent transport substrates was determined. DiOC_2_(3), dimethyl sulfoxide (DMSO), vinblastine, propidium iodide, and efflux buffer were used from the MDR1 Efflux Assay Kit (Millipore, MA, USA). After the cells were harvested and counted, they were re-suspended to 7.5 × 10^5^ cells/mL in cold (4 °C) efflux buffer (1% bovine serum albumin in RPMI-1640) containing 1 μg DiOC_2_(3). They were incubated for 15 min on ice for loading with DiOC_2_(3) and then centrifuged at 200 × *g* for 5 min. Cells were washed twice with cold efflux buffer and divided into three sample groups (2.5 × 10^5^ cells/group). Two groups were treated at 37 °C with 1 mL efflux buffer containing either DMSO or vinblastine, whereas the other group was treated with 1 mL cold efflux buffer. The two samples that were treated with vinblastine or DMSO were incubated at 37 °C for 1 h, and the other sample was incubated on ice for 1 h. Efflux termination was performed by adding cold efflux buffer. After centrifuging three times with cold efflux buffer at 200 × *g* for 5 min, the cells were resuspended in 0.5 mL ice-cold propidium iodide staining buffer. Samples were then maintained on ice until flow cytometric analysis (FACSCalibur, BD Biosciences, San Jose, CA, USA). The FL1 channel was used to measure the DiOC_2_ (3), and the FL3 channel was used to detect propidium iodide to exclude dead cells. Samples incubated on ice were used as negative controls.

For western blot analysis, cells were lysed in RIPA (radioimmunoprecipitation assay) buffer consisting of 150 mM NaCl, 50 mM Tris-HCl, 5 mM EDTA, 1% Nonidet P-40, 0.5% deoxycholate, and 1% SDS with protease inhibitor cocktail (Roche). Protein concentrations were determined with a BCA (bicinchoninic acid) protein reagent assay (Pierce, Rockford, IL, USA). Proteins were separated by electrophoresis on 8-12% SDS-polyacrylamide gels and then transferred to polyvinyl difluoride membranes. After blocking with 5% non-fat dry milk, membranes were incubated with the following primary antibodies: mouse ABCB1 (1:10000, Santa Cruz Biotechnology), rabbit ABCB1 (1:500, AbFrontier, Seoul, Korea), and mouse beta-actin (1:5000, Sigma-Aldrich, St. Louis, MO). Membranes were then incubated with peroxidase-conjugated mouse or rabbit IgG secondary antibodies (Amersham Biosciences, Arlington Heights, IL) after washing. All antibodies were diluted in Tris-buffered saline with 0.5% Tween 20. Protein bands were detected with the enhanced chemiluminescence system (Amersham Biosciences, Arlington Heights, IL), and the relative optical densities were measured with Molecular Analyst® software (Bio-Rad Laboratories, Hercules, CA, USA).

### Inhibiting MDR1 by verapamil treatment

Verapamil (20 μM; Sigma-Aldrich) was used to inhibit MDR1 at day two of differentiation in R6/2-NSC. On day 10 of differentiation, cells were fixed with 4% paraformaldehyde and stained with Em48 antibody for mHtt immunoreactivity and DAPI for counterstaining.

### Transfection of MDR1 siRNA

The dif-R6/2-NSC were transfected with MDR1 small interfering RNA (siRNA) using a pool of three target-specific 19–25 nt siRNAs (Santa Cruz Biotechnology). After two days of differentiation, cells were transfected with 100 nM MDR1 siRNA using Effectene transfection reagent (Qiagen), per the manufacturer’s protocol. siRNA-transfected dif-R6/2-NSC were evaluated on day seven. Scramble siRNA was used as a control (control siRNA).

### Reverse transcription-polymerase chain reaction (RT-PCR) for MDR1 mRNA

Total RNA from cells was extracted with TRIzol reagent (Invitrogen Life Technologies, Gaithersburg, MD), per the manufacturer’s instructions. RNA samples were reverse-transcribed using the Superscript III cDNA synthesis kit (Invitrogen Life Technologies, Gaithersburg, MD). The cDNA was amplified using a hot-start version of Taq polymerase (Takara), and primers for RT-PCR were as follows: mdr1a (sense: GAA TTG GTG ACA AAA TCG GA, anti-sense: TGT CTA TAC TGG GCT TAT TA) and GAPDH (sense: GTC GTG GAG TCT ACT GGT GT, anti-sense: TGC TGA CAA TCT TGA GTG AG). The amplification was conducted by 10 min of initial denaturation at 95 °C, followed by 32 cycles of 1 min at 95 °C, 1 min at 60 °C, and 1 min at 72 °C, and a 5 min final extension at 72 °C.

### Transfection of MDR1-GFP plasmid using Magnetofection

The pCMV6-MDR1-GFP plasmid (which encodes the open reading frame of human ABCB1 with a carboxy-terminal turboGFP tag; 10.4 kb, ABCB1-GFP) was obtained from OriGene Technologies (Rockville, MD, USA). The DNA construct was quantified using a Nanodrop spectrophotometer (NanoDrop Technologies, Thermo Scientific, Southend-on-Sea, United Kingdom). R6/2-NSC were cultured in 24 well-plates and, after two days of differentiation, cells were transfected with 0.5 μg of MDR1-plasmid using Lipofectamine LTX reagent (Invitrogen, Carlsbad, CA, USA) with 0.5 μl of CombiMAG (Chemicell, Berlin, Germany), per the manufacturer’s protocol. Cells were stained with Em48 and DAPI on day six.

### Measurement of CAG repeats

Genomic DNA samples were extracted using a QIAamp DNA mini kit (QIAgen, Germany). Mutant huntingtin gene-specific primer pairs (sense primer; 5′- CCG CTC AGG TTC TGC TTT TA -3′, antisense primer; 5′- GGC TGA GGA AGC TGA GGA G -3′) and Dr. MAX DNA Polymerase (Doctor Protein Inc, Korea) were utilised for the PCR reactions. The PCR amplification conditions were as follows: 94 °C 5 min; 94 °C 30 sec, variable temperature 30 sec, 72 °C 40 sec for 35 cycles; 72 °C 7 min. PCR products were purified using a Millipore plate MSNU030 (Millipore SAS, Molsheim, France). The purified PCR products were then Sanger sequenced with the BigDye terminator v3.1 sequencing kit and an ABI 3730xl automated sequencer (Applied Biosystems, Foster City, CA). Nucleotide sequences were determined on both strands of PCR amplification products at the Macrogen sequencing facility (Macrogen Inc., Seoul, Korea).

### Statistical analysis

Data are expressed as the mean ± SE and were analysed using Student’s *t*-test for statistical significance (P < 0.05). We used analysis of variance (ANOVA) for comparison of multiple groups. If the ANOVA was significant, Fisher’s post-hoc test was used to determine which specific groups differ significantly from one another. The data were analysed using SPSS (Statistical Package for the Social Sciences) version 17.0 (SPSS Inc., USA).

## Additional Information

**How to cite this article**: Im, W. *et al.* Multidrug resistance protein 1 reduces the aggregation of mutant huntingtin in neuronal cells derived from the Huntington's disease R6/2 model. *Sci. Rep.* **5**, 16887; doi: 10.1038/srep16887 (2015).

## Supplementary Material

Supplementary Dataset 1
