# Supplementary material for: Multidrug resistance protein 1 reduces the aggregation of mutant huntingtin in neuronal cells derived from the Huntington’s disease R6/2 model
Source: Sci Rep. 2015 Nov 20;5:16887. doi: 10.1038/srep16887 (PMC4653614; doi:10.1038/srep16887)
Supplement: Supplementary Dataset 1 [file srep16887-s1.doc]

**Multidrug resistance protein 1 reduces the aggregation of mutant *huntingtin* in neuronal cells derived from the Huntington’s disease R6/2 model**

Wooseok Im, Jaejun Ban, Jin-Young Chung, Soon-Tae Lee, Kon Chu, Manho Kim

Supplementary Data

**Supplementary Figure S1. Investigation of mHtt aggregation in R6/2 treated with verapamil.** Striatum sections of wild type, R6/2 control and R6/2 verapamil were stained with Em48 followed by DAB secondary antibody. R6/2 verapamil showed increase of mHtt aggregations staining area compared with R6/2 control. Scale bar = 10 μm.

**
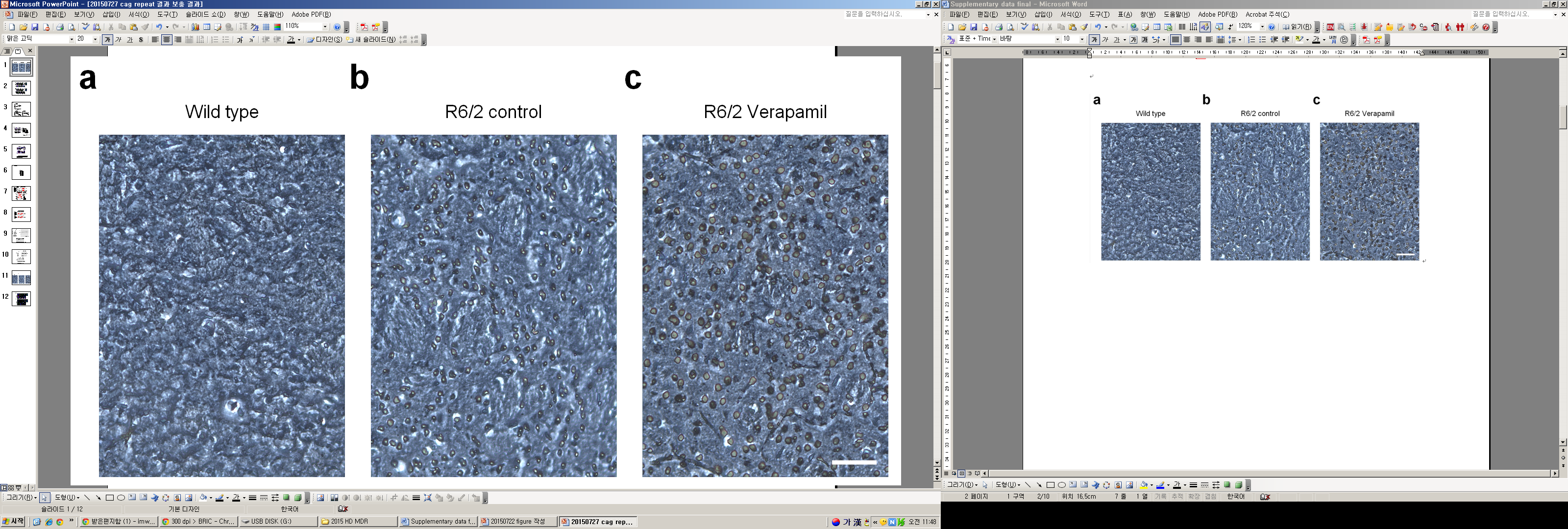
**

**Supplementary Figure S2. The effect of MDR1 overexpression on aggregation of mHtt by MDR1-GFP plasmid.** dif-R6/2-NSC were transfected with GFP or MDR1-GFP plasmid. Immunostaining results showed that co-localization (34.3 ± 2.5%) of Em48 and GFP of total GFP-expressing cells in cells transfected with GFP plasmid (a). On the other hand, MDR1-GFP was almost not co-localized with Em48 staining of total GFP-expressing cells in cells transfected with MDR1-GFP plasmid (b). (Blue: DAPI, Red: Em48, Green: GFP)


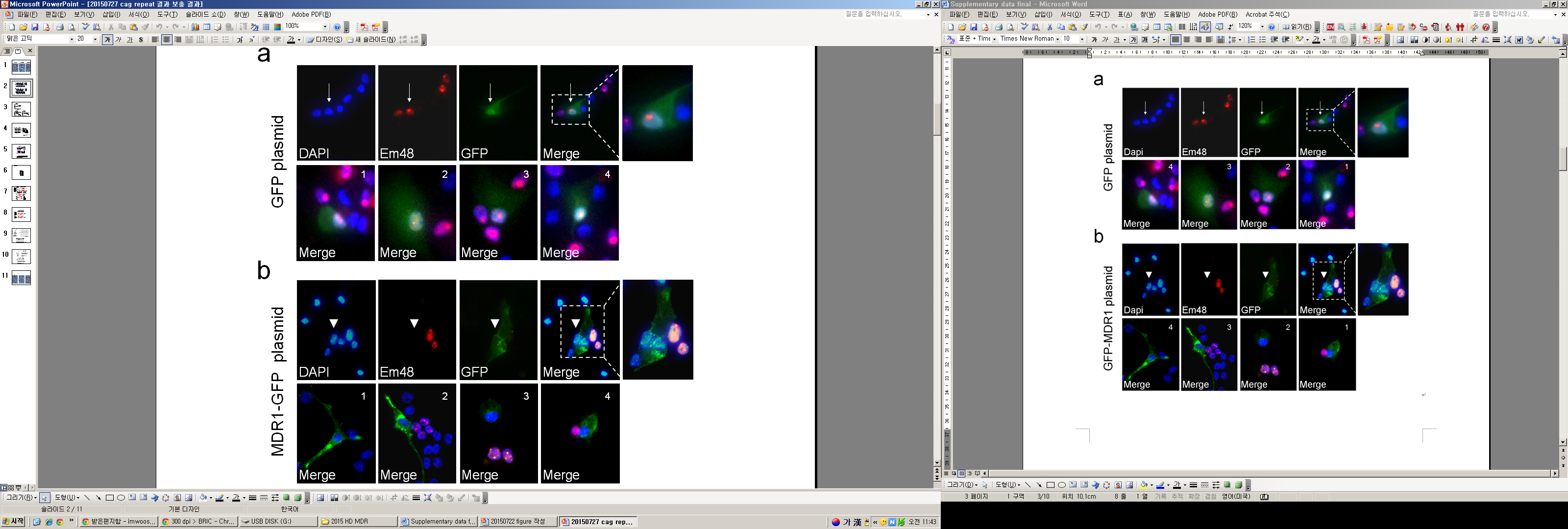


**Supplementary Figure S3. Measurement of body weight and rotarod test.** (a) Result of body weight measurement of wild type, R6/2 vehicle (n=4, 2 males and 2 females) and R6/2 treated with verapamil (n=4, 2 males and 2 females) showed similar body weight between groups. R6/2 vehicle vs R6/2-verapamil: *F*_2,9_ = 0.12, *P =* 0.88 at 5 weeks; *F*_2,9_ = 0.03, *P =* 0.97 at 6 weeks; *F*_2,9_ = 0.001, *P =* 0.99 at 7 weeks; *F*_2,9_ = 0.02, *P =* 0.98 at 8 weeks; *F*_2,9_ = 0.04, *P =* 0.96 at 9 weeks. Results of fall latency (b) and body weight (c) of wild type, R6/2 vehicle and R6/2 treated with rifampin (each n=4, 4 males). R6/2 vehicle vs R6/2 rifampin for fall latency: *F*_1.6_ = 4.39, *P =* 0.08 at 7 weeks; *F*_1.6_ = 1.04, *P =* 0.35 at 8 weeks; *F*_1.6_ = 0.03, *P =* 0.87 at 9 weeks; *F*_1.6_ = 0.55, *P =* 0.48 at 10 weeks; *F*_1.6_ = 0.38, *P =* 0.56 at 11 weeks; *F*_1.6_ = 1.56, *P =* 0.26 at 12 weeks. R6/2 vehicle vs R6/2 rifampin for weight: *F*_1.6_ = 9.4, *P =* 0.02 at 7 weeks; *F*_1.6_ = 1.4, *P =* 0.28 at 8 weeks; *F*_1.6_ = 0.79, *P =* 0.41 at 9 weeks; *F*_1.6_ = 1.34, *P =* 0.29 at 10 weeks; *F*_1.6_ = 2.10, *P =* 0.20 at 11 weeks; *F*_1.6_ = 4.74, *P =* 0.07 weeks.

**
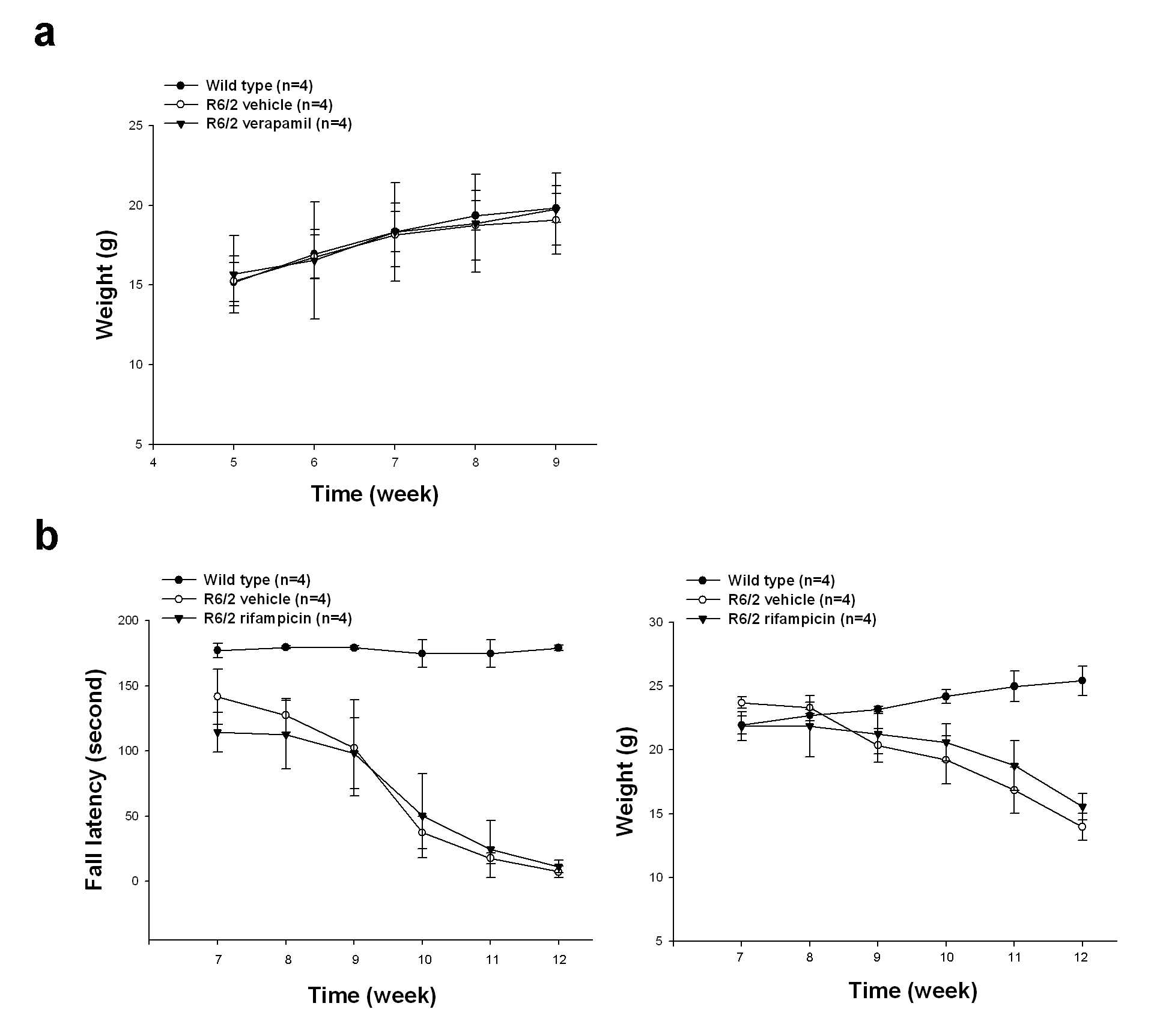
**

**Supplementary Figure S4. Immunohistochemistry in brain of R6/2 treated with vehicle (DMSO) or rifampin**. Arrows indicate smeared red fluorescence around condense spots (red) in nucleus, whereas there are mainly just blue (arrowheads) around these in nucleus of R6/2-Rifampin. (Blue: DAPI, Red: Em48)

**
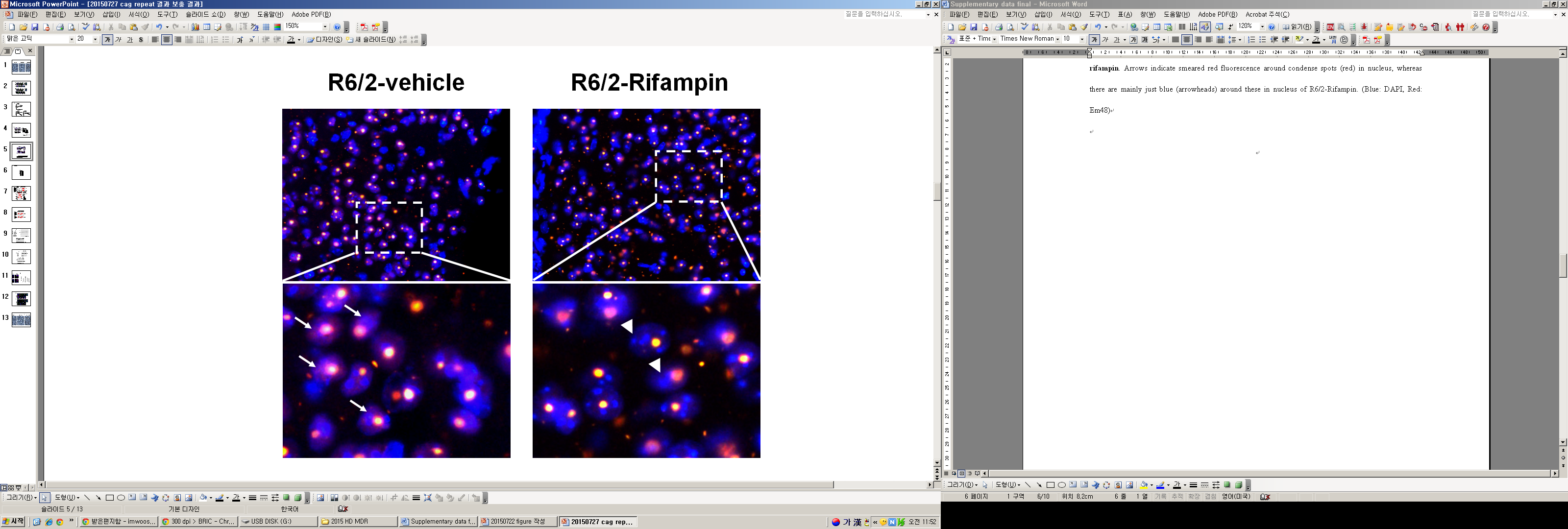
**

**Supplementary Figure S5.** **The effect of MDR1 inhibitor by on aggregation of mHtt by verapamil depending of the time.** R6/2-NSC were treated with verapamil at 3, 5, and 7 days after differentiation. (a) Differentiated cells were stained with Em48 and DAPI at 10 days. (b) Verapamil treatment at 3 and 5 days (D3 and D5) showed increased Em48 staining compared with control (D3 > D5 > control) whereas there was no significant difference between control and 7 days group (D7). (Control: 31.3 ± 1.9%, D3: 44.4 ± 3.2, D5: 37.5 ± 1.7%, D7: 33.8 ± 0.8), * P < 0.05, Scale bar = 20 μm

**
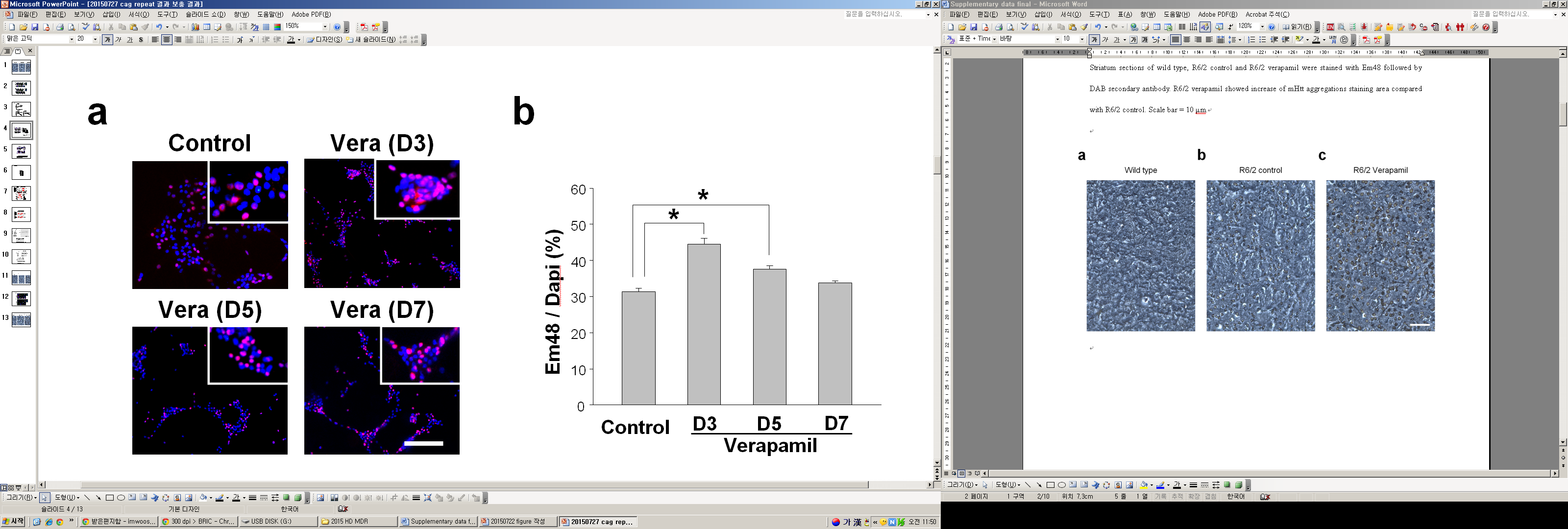
**

**Supplementary Figure S6. The measurement of CAG repeats in R6/2 vehicle or verapamil groups.** R6/2-vehicle and -vera groups showed each 120.0 ± 3.7 and 118.4 ± 4.5 CAG repeats. NS=non-significant.

**
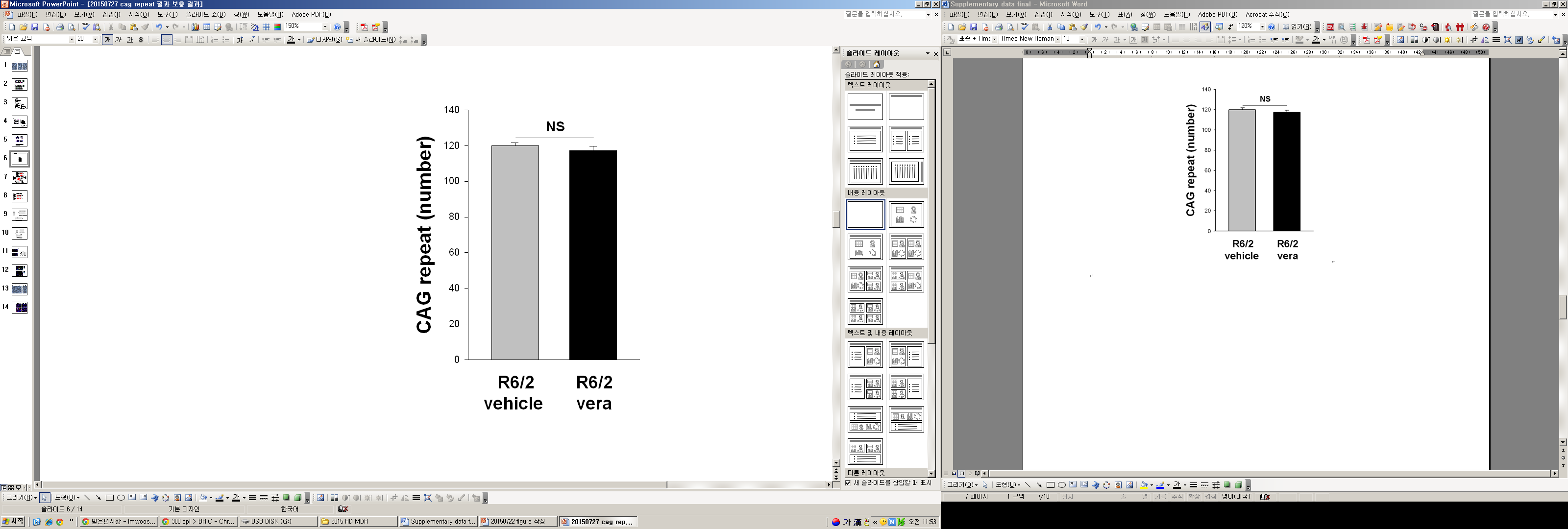
**

**Supplementary Figure S7. The full length blots and dot blots results.** MDR1 expression was investigated between dif-R6/2-NSC and R6/2-NSC (a), and in dif-R6/2-NSC transfected with control-siRNA or MDR1-siRNA (b). Accumulation of mHtt aggregates was measured in R6/2 mice treated with rifampin by western blot using the gradient (4-12%) running gel (c) and the 5% running gel (d). Accumulation of mHtt aggregates in brain of R6/2 mice was detected (e) and decrease of it in R6/2 mice treated with rifampin was confirmed (f and g) by dot blot. *P<0.01

**
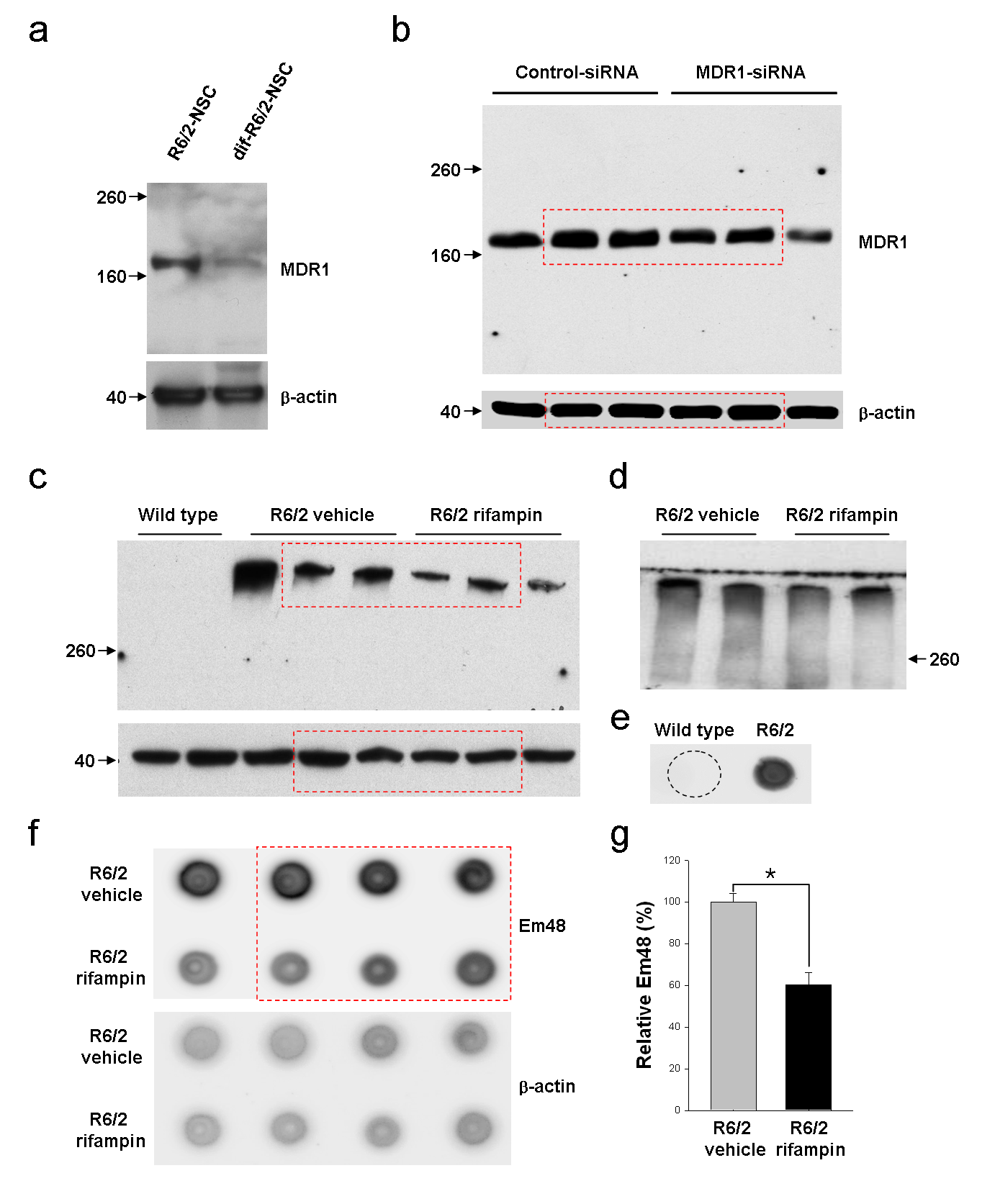
**

**Supplementary Method**

**Measurement of CAG repeat.** Genomic DNA samples were extracted using a QIAamp DNA mini kit (QIAgen, Germany). Mutant Huntingtin gene specific primer pairs (sense primer; 5'- CCG CTC AGG TTC TGC TTT TA -3', antisense primer; 5'- GGC TGA GGA AGC TGA GGA G -3') and Dr.MAX DNA Polymerase (Doctor protein INC, Korea), were utilized for the PCR reactions. PCR amplification conditions were as follows: 94°C 5 min; 94°C 30 sec, variable temperature 30 sec, 72°C 40 sec for 35 cycles; 72°C 7 min. PCR products were purified using Millipore plate MSNU030 (Millipore SAS, Molsheim, France). The purified PCR products were then Sanger-sequenced with the BigDye terminator v3.1 sequencing kit and a 3730xl automated sequencer (Applied Biosystems, Foster City, CA). Nucleotide sequences were determined on both strands of PCR amplification products at the Macrogen sequencing facility (Macrogen Inc., Seoul, Korea).

**Western blot with the gradient gel.** Protein extracts from the brains of R6/2 mice were prepared using RIPA buffer (Thermo, USA) with freshly added protease inhibitor and phosphatase inhibitor (Roche, USA). Then, 30 µg of protein samples were separated by 4-12% gradient SDS-PAGE (NuPAGE Novex, Invitrogen, Carlsbad, CA) for two hours and electrophoretically transferred to polyvinylidene difluoride (PVDF, Millipore Corporation, Bedford, MA, USA) for three hours. The membranes were blocked in 5% skim milk in 0.05% Tween 20/Tris-buffered saline (TBS-T) and incubated with anti-Em48 (MAB5374, 1:500, Millipore Corporation, Bedford, MA, USA) overnight at 4 °C. Then, the membranes were washed three times in TBST and incubated for two hours with horseradish peroxidase-conjugated secondary antibody (dilution 1:3,000). Immunoreactive proteins were detected with enhanced chemiluminescence reagents (Advansta, Menlo Park, CA, USA).

**Dot blot assay.** The dot blot assay for the detection of accumulated mHtt aggregates was performed according to the published protocol (20190739) using the anti-Em48 antibody. Briefly, dot blots were prepared by blotting 10 μg of total proteins onto nitrocellulose membranes (Invitrogen Life Technologies, Carlsbad, CA, USA) and were processed as described for western blots after drying the membrane. The membrane was blocked for one hour at room temperature with TBST (5% nonfat milk in Tris-buffered saline containing 0.05% Tween 20) and probed with anti-Em48. A horseradish peroxidase-conjugated anti-mouse secondary antibody (Vector Laboratories, Burlingame, CA, USA) was used at 1:3,000 for two hours at room temperature. Blots were detected using ECL chemiluminescence detection reagent (Advansta, Menlo Park, CA, USA). Images were obtained by exposing the membranes to Hyperfilm MP (ImageQuant LAS 4000 Mini, GE Healthcare).
